# A case analysis of a mass treatment approach to control GI and water-related conditions in Sudan

**DOI:** 10.1186/s12889-021-12154-0

**Published:** 2021-11-17

**Authors:** Alaaddin Salih, Mohamed Mohamed

**Affiliations:** 1 National Academy of Health Sciences, Khartoum, Sudan; 2 University of Gezira, Wad Madani, Sudan

**Keywords:** Research challenges, Strategies, Emergency settings, Security, Collaboration, Community engagement

## Abstract

**Background:**

The efficiency of mass chlorination in controlling diarrheal diseases during acute emergencies has been extensively reported in literature. However, long-term crises received unparallel attention. Researchers have previously carried out a trial that investigated the impact of using chemical means to treat water reservoirs of Um-Baddah Nevachah, a refugee camp located in the western outskirts of Khartoum, Sudan.

A double-blind community experimental trial was carried out by randomly adding either chlorine or a placebo to the major water tanks in the area. Data were collected using a mixed-methods study design. The primary input was the quantitative data derived from total coliforms lab tests and records of the local primary healthcare center, while the embedded (nested) portion generated most of the qualitative data from direct face-to-face interviews.

As a case analysis, this study aims to critically appraise the original trial. In the Background the authors discussed the context of the trial, approach used, and outcomes. Discussion section included three issues related to the trial: scientific importance, challenges and strategies.

**Discussion:**

Importance: There are two factors that contribute to the importance of this study: First, the integrated and systematic approach followed to resolve associated challenges. The study swiftly moved from investigating potential water contamination, to test whether it is related to an endogenous focus that auto-taints drinking water, and finally it explored the impact of tanks chlorination on public health. Second, the longstanding humanitarian context which remains largely underreported in literature. Challenges: funding limitations were among the first obstacles faced. During the fieldwork preparation phase, a lot of work was required to resolve logistical and security challenges. Keeping volunteers motivated was the biggest concern during the last phase of data collection. Strategies: The “Matrix Solutions Strategy” was developed and used to optimize scarce resources to simultaneously target multiple problems through a single intervention.

**Conclusion:**

Key lessons learned from the whole experience were: persistence is paramount for the success of studies in precarious situations; lateral thinking generates alternative solutions that are novel, feasibility and practical in resources-limited settings; and finally respecting local culture and regulations is essential for building trust with both authorities and vulnerable societies.

## Background

Armed conflicts have been ripping Sudan apart for decades. War in the former southern part of the country is often dubbed as the longest war in Africa that outbroke in 1955 [1]. Darfur region, in the western part of Sudan, has had its fair share of natural and manmade disasters, like the severe drought that struck the region during the 1980’s and the notorious bloodshed that had been ongoing for over two decades by now. Over the years, millions of painfully afflicted people have ventured off to seek refuge elsewhere within Sudan, only to find it at last in squalid settlements [2].

Diarrheal diseases remain one of the leading causes of mortality during emergencies worldwide. Chlorination has been in use for a long time to control acute diarrhea. Efficiency of this approach has been the research question for many humanitarian studies including ours. Those studies casted light on acute crises at the expense of extended ones. Therefore, inadequate attention has hitherto been directed towards assessing value of chlorination in long-term settings compared to emergencies. To better understand the former, a double-blind mixed methods community experimental trial was carried out. Sources of primary quantitative data were the total coliforms lab results and records of the local primary health center, whereas face-to-face interviews with some inhabitants accounted for the embedded or nested part (qualitative).

The main purpose of this case analysis is to provide a critical appraisal of the original trial and to examine the following 4 aspects: its scientific importance, the circumstances in which it was carried out, the challenges that investigators encountered, and finally the solutions and strategies they came up with.

Um-Baddah Nevachah refugee camp was selected to investigate claims of widespread waterborne diseases. More specifically, the study examined the effects of treating tank water in an internally displaced people (IDP) camp. It has been in place for more than 15 years and is located in the western suburbs of Khartoum, Sudan, 30 km from the city center. The camp spans a 9.8 km^2^ total area divided into six residential quarters. The total population is over 9000 from 57 tribes. The vast majority live under low socioeconomic status with no access to basic infrastructure. Available governmental services include a single primary healthcare center and four basic schools. There are no central grids to supply either electricity or water, however, the latter is delivered to households on donkey-drawn wheeled barrels known locally as “karrow”. The only drinking water sources are four wells (average depth = 260 m) [3].

The first step in the original trial [3] was to assess wells water. Based on contamination status the four wells were divided into two equivalent case and control groups. Efficiency of the intervention was evaluated based on before-and-after comparisons of the four data collection methods used, viz., total coliforms lab results, questionnaires, face-to-face interviews, and medical records.

## Discussion

### The scientific importance of this research

The value of the original trial is derived from (1): its approach; and (2) the setting of the study. In addition, investigators had to adapt to a challenging local context and be innovative with the strategies they came up with to tackle these obstacles, without compromising scholarly standards nor principles of responsible conduct. It is also important to highlight a totally different aspect of the study, which is its social impact. For the first time, our study population enjoyed a better quality of life after years of experiencing the toll of water contamination on their own health and environment.

Although mixed-methods research is well established in literature [4–7], to the best of our knowledge our trial was the first to use that methodology in Sudan. The main objective of the original trial was to demystify the root causes behind the widespread diarrheal diseases in that disadvantaged population. Because of the complexity of the problem an unconventional approach had to be adopted. That is to go beyond answering simple and direct questions like studying epidemiology of common water-borne diarrheal diseases in the area, or elucidating impact of chlorination on pathogens present in the area’s tainted water. Rather, there was a consensus on taking a holistic approach that simultaneously addresses all of these questions.

It is believed that about 80% of diseases in developing countries are water-related [8]. For such prevalent illnesses that are associated with heavy morbidity, and yet largely preventable, it was quite unclear why those underdeveloped countries have not widely adopted a simple, cost-effective, and highly efficient approach as chlorination [9, 10] that has been around for decades. This is pretty much in line with the findings of Eltahir et al. [11] study that was the first of its kind to investigate the outcome of treating drinking water with chlorine in IDP and refugees camps in Sudan.

During one of the focus group discussions, research team became aware of the successful chlorination interventions that local tanks underwent in the past. The biggest question was why water got contaminated again a short while afterwards? To address this questions researchers decided to work on two parallel lines: First, assessing supply chain of the drinking water all the way from source to mouth, and Second, evaluating impact of contamination on residents’ health through records of local health center. Upon inspecting tanks, they were found to be archaic, rusty and without sealed covers. Based on these observations, presence of contaminants within tanks was hypothesized. The team suspected a potential source of contamination was a fabricated radiator-like system that cools down the well pump by means of heat exchange. It works by taking water out the tank, pushing it into pipes that surround the pumping machine, and finally returns hot outflow back into the tank to mix up with drinking water.

Another factor that made our original study particularly important was the context of longstanding crisis in which it was carried out. As time goes by, unsettled conflicts becomes even more complicated by occasional events like public health emergencies, for instance. Although Um-Baddah Nevachah settlement was established decades ago, the area has never been part of the purview of the governmental agencies nor that of the non-governmental organizations (NGOs). As a result, local residents remained without access to the much-needed public health services. Such negligence brings no solutions to conflicts transforming them into “chronic” issues. More studies are needed to decipher the intricacies of such understudied context of complicated longstanding disputes.

Outbreaks of water-borne diarrheal illnesses during acute emergencies are known public health risks. Globally, this has been effectively managed by treating water with chlorine in accordance with World Health Organization (WHO) guidelines [12]. However, in longstanding crises the nature of contaminants might slightly change, jeopardizing the efficiency of chlorine as a disinfectant. Therefore, identifying the source of contamination is critical when investigating similar circumstances, as noted in the research team’s recent publication [3]. In a nutshell, during longstanding disputes, more complex mechanisms could taint drinking water. Unlike the case during emergencies, it is important to understand these causes first before going ahead and treating water with chlorine.

### Challenges to research

#### Lack of funding

In an absence of national research funding programs, the research team lacked access to domestic grants and faced numerous barriers vying for international awards. Regardless, researchers had to resort to the meager research budget to fund early stages of the study and were thereby able to scrape by. Later, the team fortunately landed an award that complemented the research budget.

#### Logistics

Logistics had always been a challenge throughout the course of the original study, and it ranged from organizational matters to telecommunication problems.

Before embarking on practical and data collection phases, investigators had to go through a tedious and time-consuming process to collect necessary approvals and clearances. In addition to Ethical Clearance from the Institutional Review Board (IRB), local community representative, known as People’s Committee or Quarters Popular Committee (QPC), requested further letters.

Communication limitations constituted a major obstacle to our project. With the camp lacking a reliable mobile network coverage, it was difficult to get in touch with our partners and research assistants inside the camp while investigators were away. Needless to say that internet-based services and modalities like emails, messaging, instant chat applications, and Voice over Internet Protocol (VoIP) apps were not options either. Apart from the inconvenience of frequently visiting the area, telecommunication obstacles had no substantial impact on the integrity of our field operations that include recruitment and data collection.

#### Security

Security was certainly one of the major concerns in our case. Many outlaws and criminals were active in the camp and adjacent areas. To stay clear from potential dangers, research team decided to make all expeditions during daylight, which tightened time constraints even further. At the core of investigators’ personal safety was their ability to work their way amidst the high tensions between local authorities and camp refugees without compromising trust of either. Right from the beginning, and during the first field visit, researchers realized that as soon as they passed by the large tactical police camp next to the study area, they were been stalked by security and intelligence personnel. Living with such measures was the only way to get the study done. On a side note, this example demonstrates how investigators gave the trust-building process the time it needs, which combined with their multiple data collection methods (lab results, questionnaires, face-to-face interviews and local health center records) both represent the core recommendations made by Vincent Manirakiza [13] for researchers working in post-conflict milieus of mistrust.

#### Data collection

Data collectors were recruited on an exclusively voluntary basis. Offering some compensatory rewards was our way to recognize volunteers’ participation and to create some momentum among them. During our first meeting with data collectors, we made it crystally clear that they were not entitled to receive any financial incentives because of the tight budget the study was run on. Instead, investigators suggested acknowledging their input through certificates and endorsement letters. They were fine with that arrangement.

After initially accepting these terms and when collected data were due for submission to investigators, research assistants have changed their minds and started demanding some pecuniary rewards. Budget constraints made such request unattainable. Therefore, primary investigator developed an alternative strategy based on two points:

- Clearly communicating researchers doubts about delivering on an initial promise to submit the final study report to the Head of People’s Committee, had they not provided the team with the data needed to write up the study. QPC members were keen to collect that report to support their claims for funding.
- Data collectors were volunteers with prominent political motivations. Therefore, researchers leveraged the involvement of key political leaders to act as mediators between the research team and the volunteers. This helped to resolve the situation to the benefit of all parties.

### Research strategies

#### Matrix solutions strategy

Matrix Solutions Strategy is a model designed by the investigators to deal with multiple problems all at once using available resources. For instance, insufficient funding, lack of substantial means of transportation, and security concerns, were among the challenges discussed earlier during the preparatory phase. The possible matrix solution that investigators came up with was to commute together in one of their private vehicles.

#### Civic engagement approach “Judiaa”

“Judiaa” is a term used for traditional approaches of mediation in Sudan. The way it is traditionally practiced is by getting a bunch of impartial, affluent and trusted civic leaders to engage in lengthy liaisons between the parties. To conduct the study, researchers had to operate in a highly polarized environment with parties of conflicting interests.

The study area was under strict control of the Sudanese security forces and it was inhabited by internally-displaced Darfuris. Tensions between both sides became obvious soon as researchers wrapped up their first meeting with local residents. Therefore, researchers had to come up with a strategy to avoid a standoff with authorities without violating community trust. At the core of that strategy was approaching the government-backed People Committee simultaneously with the Native Administration which represents locals. The meeting concluded that the Committee should contact the official side, while the Administration was asked to publicize the study mission in the area. This approach did not only help navigating the nexus between official requirements and community trust, but also it has created a perpetual public impact through the newly found common ground.

#### Cultural adaptation

To successfully implement a community-based experimental trial, considerable cultural adaptations had to be made by the group working in the field. Principal investigators in that study adopted a culturally sensitive approach that respects indigenous norms and rituals. Even the tiniest details have been catered for, from the traditional greeting style of tri-hands shakes, to sitting on the ground in a cross legged posture, and finally accepting locals invitations to have meals or drinks with them, even when the served food or drink is inconvenient.

#### Capacity building

This strategy concerns the last of the four challenges stated earlier. Running a data collection scheme on a limited budget calls for alternative solutions. Rather than hiring and paying research assistants from our institution, study leaders opted to train data collectors from the area. Although that meant considerable pecuniary and time resources had to be invested to train those volunteers, the approach was absolutely successful in terms of societal capacity building.

This approach replaced cash rewards with various types of incentives. Examples include: equipping recruitees with basic research skills; creating a rigorous academic experience in which trainees find themselves fully immersed in research; offering internship opportunities to boost trainees resumes and make them more competitive and marketable; and crediting participants with certificates and references to promote themselves.

## Conclusions

Invaluable lessons were learned throughout the course of our original trial project, which made it a great educational experience as much as a research one. So often have the investigators heard about difficulties of conducting a research in such challenging settings, but it is fair to tell that never before this study have they recognized these difficulties fully. Without the adequate means and wherewithal, becoming adaptive and innovative was the best way to get things done. It took the team a ton of persistence and determination to successfully endeavor that challenging research project. In fact, researchers had to use different approaches that appealed to each of the stakeholders involved so as to have all of them onboard.

Perhaps, the most important take home message is to always exhaust all resources to devise homegrown solutions that are likely to pay off. Better than toying with some fancy ideas or waiting for conditions to change, one should seriously buckle down for smarter alternatives. For instance, investigators went around the funding challenge by: paying out of pocket to kick start the trial, tightening up research budget, and applying for different potential grants. Concerning safety, our strategy had three anchors: commuting on shared rides, showing up in the camp during daytime, and complying with authorities requests.

Another key point to remember is realizing the hazards associated with research in general. It seems that research, by nature, carries some degree of risk in most cases. This could be as clear as catching up a contagion, like nCoV-2 of COVID-19, in case of wet lab research. However, there are other types of research that are associated with more serious hazards. Examples include research in unstable and precarious settings where personal safety and even lives of investigators could be under direct threat. One way to manage such risks is by creating a risk matrix that sets out potential menaces, their sources, and their means of mitigation.

Last but not the least, throughout the process researchers have kept in mind how imperative it is to abide by local regulations and respect traditional culture. They believe that cultural humility and appreciation of community ethos both open up hearts and bridge gaps between scholars and community members [14, 15]. During crises, people often find themselves in fragile situations, to which they may react in different ways. Some might be skeptical of those who prioritize researching over providing the much needed rescue and aid efforts during such critical times and circumstances [16]. The best way to respond to such trepidations is through transparent dialogue that respects people’s concerns.

## Data Availability

Data sharing is not applicable to this article as no datasets were generated or analyzed during the current study.

## Abbreviations

GI: Gastrointestinal “Tract”
IDP: Internally Displaced People
IRB: Institutional Review Board
NGOs: Non-Governmental Organizations
QPC: Quarters Popular Committee
WHO: World Health Organization
